# Optical constraints on two-photon voltage imaging

**DOI:** 10.1117/1.NPh.11.3.035007

**Published:** 2024-08-13

**Authors:** F. Phil Brooks, Hunter C. Davis, J. David Wong-Campos, Adam E. Cohen

**Affiliations:** Harvard University, Department of Chemistry and Chemical Biology, Cambridge, Massachusetts, United States

**Keywords:** two-photon, voltage imaging, shot noise

## Abstract

**Significance:**

Genetically encoded voltage indicators (GEVIs) are a valuable tool for studying neural circuits *in vivo*, but the relative merits and limitations of one-photon (1P) versus two-photon (2P) voltage imaging are not well characterized.

**Aim:**

We consider the optical and biophysical constraints particular to 1P and 2P voltage imaging and compare the imaging properties of commonly used GEVIs under 1P and 2P excitation.

**Approach:**

We measure the brightness and voltage sensitivity of voltage indicators from commonly used classes under 1P and 2P illumination. We also measure the decrease in fluorescence as a function of depth in the mouse brain. We develop a simple model of the number of measurable cells as a function of reporter properties, imaging parameters, and desired signal-to-noise ratio (SNR). We then discuss how the performance of voltage imaging would be affected by sensor improvements and by recently introduced advanced imaging modalities.

**Results:**

Compared with 1P excitation, 2P excitation requires $∼10^{4}$-fold more illumination power per cell to produce similar photon count rates. For voltage imaging with JEDI-2P in the mouse cortex with a target SNR of 10 (spike height to baseline shot noise), a measurement bandwidth of 1 kHz, a thermally limited laser power of 200 mW, and an imaging depth of >300  μm, 2P voltage imaging using an 80-MHz source can record from no more than ∼12 neurons simultaneously.

**Conclusions:**

Due to the stringent photon-count requirements of voltage imaging and the modest voltage sensitivity of existing reporters, 2P voltage imaging *in vivo* faces a stringent tradeoff between shot noise and tissue photodamage. 2P imaging of hundreds of neurons with high SNR at a depth of >300  μm will require either major improvements in 2P GEVIs or qualitatively new approaches to imaging.

## 1 Introduction

A long-standing dream in neuroscience has been to record the membrane potential of hundreds or thousands of neurons simultaneously in a behaving animal. Such measurements could reveal functional connections, probe input–output properties of cells and microcircuits, and help discern principles of neural computation. Recent advances in genetically encoded voltage indicators (GEVIs) have substantially improved their signal-to-noise ratio (SNR), enabling recordings from dozens of cells in superficial tissue using one-photon (1P) imaging.^1^^–^^3^ There have also been improvements in instrumentation^4^^,^^5^ and reporters^6^ for two-photon (2P) voltage imaging, enabling voltage imaging at depths up to 500  μm, though the number of simultaneously recorded cells at this depth remains <3. Most applications of voltage imaging *in vivo* have been with 1P excitation,^7^^–^^10^ whereas for ${\mathrm{Ca}}^{2+}$ imaging, 2P microscopy is the dominant approach.^11^ This raises the question: What are the scaling properties and relative merits of 1P versus 2P voltage imaging *in vivo*? How can a researcher considering a voltage imaging experiment decide which approach to use?

The physical requirements of ${\mathrm{Ca}}^{2+}$ imaging and voltage imaging differ substantially, so intuitions may not transfer. For ${\mathrm{Ca}}^{2+}$ imaging, typical events last 100 to 500 ms and have amplitudes of ΔF/F∼100%. Signals come from the bulk cytoplasm. For voltage imaging, action potentials last ∼0.3 to 2 ms and typically have amplitudes of ΔF/F∼10% to 30%, though subthreshold events can be 100-fold smaller. Signals are localized to the cell membrane. Thus, the key challenge in voltage imaging is to acquire adequate SNR and imaging speed in the presence of shot noise and motion artifacts while maintaining tissue-safe laser powers.

Here, we explore, with the support of modeling and data from representative voltage indicators, how molecular and optical parameters affect the balance among SNR, number of simultaneously recorded cells, and tissue damage. Many of the arguments about the scaling of noise^12^^,^^13^ and 2P signal^14^^,^^15^ are found elsewhere in the literature, but with the recent publications seeking to reach^5^^,^^6^^,^^16^^–^^22^ or transcend^23^^–^^27^ these limits, we believe that consolidation of the arguments with a specific application to voltage imaging is warranted.

Our results support the preference for 1P over 2P imaging at shallow depths and the use of 2P voltage imaging at depths where 1P recordings are inaccessible due to light scattering. However, at depths beyond the 1P limit, 2P voltage imaging signals are severely constrained by thermal and shot noise limits. We address the potential of advanced instrumentation and analysis techniques to improve performance beyond current limits.

## 2 Materials and Methods

### 2.1 Human Embryonic Kidney (HEK) Cell Culture

HEK293T cells were maintained at 37°C, 5% ${\mathrm{CO}}_{2}$ in Dulbecco’s Modified Eagle Medium (DMEM) supplemented with 10% fetal bovine serum, 1% GlutaMAX-I, penicillin (100  U/mL), and streptomycin (100  mg/mL). For maintaining or expanding the cell culture, we used a 35 mm tissue-culture-treated culture dish (CorningWare, Corning, New York, United States). For each imaging experiment, cells in one 35 mm dish were transiently transfected with the construct to be imaged using polyethyleneimine (PEI) in a 3:1 PEI-to-DNA mass ratio. For all the imaging experiments, cells were replated on glass-bottomed dishes (Cellvis, D35-14-1.5-N, Mountain View, California, United States) 36 h after transfection. Imaging was performed ∼6  h after replating. Before optical stimulation and imaging, the medium was replaced with extracellular (XC) buffer containing 125 mM NaCl, 2.5 mM KCl, 3 mM ${\mathrm{CaCl}}_{2}$, 1 mM ${\mathrm{MgCl}}_{2}$, 15 mM HEPES, and 30-mM glucose (pH 7.3). For BeRST1 experiments in HEK cells, cells were stained with 1  μM BeRST1 for 30 min prior to 3× wash with XC buffer before imaging.

### 2.2 Microscope and Illumination Control

All imaging was performed using the Luminos bi-directional microscopy control software^28^ on a custom-built upright microscope equipped with 1P and 2P illumination paths and a shared emission path to a scientific complementary metal oxide semiconductor (sCMOS) camera (Hamamatsu, ORCA-Flash 4.0 v2, Bridgewater, New Jersey, United States). The 1P illumination path contained a 488 nm laser (Coherent OBIS, Saxonburg, Pennsylvania, United States), a 532 nm laser (Laserglow LLS-0532, North York, Canada), and a 635 nm laser (Coherent OBIS). The outputs of the 488 nm and 532 nm lasers were modulated using a multichannel acousto-optic tunable filter (Gooch & Housego PCAOM NI VIS driven by G&H MSD040-150, Ilminster, United Kingdom). The 635 nm laser was modulated using its analog modulation input and an external neutral density filter wheel (Thorlabs, Newton, New Jersey, United States). The 488 nm and 532 nm lasers were patterned via a digital micromirror display (Vialux V-7001 V-module, Chemnitz, Germany). To convert illumination intensity to power per cell, we approximated HEK cells as circles with a 10  μm diameter, e.g., an intensity of $1\;W/{\mathrm{cm}}^{2}$ corresponded to 0.8  μW per cell.

The two-photon illumination path comprised an 80 MHz tunable femtosecond laser (InSight DeepSee, Spectra Physics, Milpitas, California, United States), an electro-optic modulator (ConOptics 350-80-02, Danbury, Connecticut, United States) and two galvo mirrors for steering (Cambridge Technology 6215H driven by 6671HP driver). Power calibration was performed with a Thorlabs PM400 power meter with a photodiode-based sensor (S170C) and a thermal sensor (S175C) for 1P and 2P illumination, respectively. Electrical stimuli and measurements were performed using a National Instruments 6063 PCIe DAQ. All imaging was performed with a 25× water immersion objective [Olympus XLPLN25XWMP2, Tokyo, Japan, 2 mm working distance, numerical aperture (NA) 1.05]. At each wavelength, the dispersion was adjusted to maximize the 2P fluorescence signal. The wavelengths for 2P excitation of QuasAr6a and BeRST1 were chosen to drive the $S_{0}$ to $S_{2}$ transition rather than the $S_{0}$ to $S_{1}$ transition because the $S_{2}$ transition was stronger.

### 2.3 Measuring Voltage-Sensitive Fluorescence *In Vitro*

All imaging and electrophysiology experiments were performed in an XC buffer. Concurrent whole-cell patch clamp and fluorescence recordings were acquired on the microscope described above. Filamented glass micropipettes were pulled to a tip resistance of 5 to 8 MΩ and filled with an internal solution containing 125 mM potassium gluconate, 8 mM NaCl, 0.6 mM ${\mathrm{MgCl}}_{2}$, 0.1 mM ${\mathrm{CaCl}}_{2}$, 1 mM EGTA, 10 mM HEPES, 4 mM Mg-ATP, and 0.4 mM Na-GTP (pH 7.3), adjusted to 295 mOsm with sucrose. Whole-cell patch clamp recordings were performed with an Axopatch 200B amplifier (Molecular Devices, San Jose, California, United States). Fluorescence was recorded in response to a square wave from −70 to +30  mV.

### 2.4 In-House AAV Packaging

AAV2/9 JEDI-2P vectors were packaged in-house based on a published protocol.^29^ Briefly, ∼50% to 70% confluent HEK293T cells grown in DMEM supplemented with 5% FBS were triple transfected with pHelper, pAAV ITRexpression, and pAAV Rep-Cap plasmids using acidified (pH 4) PEI (DNA-to-PEI ratio 1:3) in ∼1 to 2 T175 flasks ($∼2\times 10^{7}\;\text{cells}/\text{flask}$). The adeno-associated virus (AAV)-containing medium was harvested on day 3, and the AAV-containing medium and cells were harvested on day 5. For the second harvest, AAVs were released from the cells with citrate buffer (55 mM citric acid, 55 mM sodium citrate, and 800 mM NaCl, 3 mL per flask). The two harvests were then combined and precipitated with PEG/NaCl (5×, 40% PEG 8000 (w/v), 2.5 M NaCl, 4°C overnight). The low-titer virus was then purified with chloroform extraction [viral suspension and chloroform 1:1 (v/v)], aqueous two-phase partitioning [per 1 g of the AAV-containing supernatant, add 5 g of 20% $({\mathrm{NH}}_{4}{)}_{2}{\mathrm{SO}}_{4}$ solution and 1.5 g of 50% PEG 8000 solution], and iodixanol discontinuous gradient centrifugation (15%, 25%, 40%, and 54% iodixanol gradient prepared from OptiPrep] [60% (w/v) iodixanol, Axis-Shield PoC AS, Dundee, United Kingdom]. The purified AAV titer was determined via quantitative polymerase chain reaction (SYBR Green, primer for forward ITR: 5′-GGAACCCCTAGTGATGGAGTT-3′; primer for reverse ITR sequence 5′-CGGCCTCAGTGAGCGA-3′).

### 2.5 *In Vivo* Imaging

All animal experiments were approved by the Institutional Animal Care and Use Committee of Harvard University. The cranial window surgery for *in vivo* imaging was based on previously published protocols.^7^ Briefly, an adult CD1 mouse was injected with a 50-nL viral mix in four sites in the whisker barrel cortex at 100, 200, 300, 400, and 500  μm below the tissue surface. The viral mix had a final concentration of $5\times 10^{12}\;\mathrm{vg}/\mathrm{mL}$ pAAV-EF1a-DIO-JEDI-2P-Kv2.1motif and $1\times 10^{11}$ CamKII-Cre in AAV 2/9. A cranial window and mounting plate were then installed over the injection sites. Two weeks after surgery and injection, a head-fixed CD1 mouse was imaged at 1.5% isoflurane with the dose adjusted to maintain a stable breathing rate. The mouse was kept on a heating pad (WPI ATC2000) to maintain a stable body temperature at 37°C, and its eyes were kept moist using ophthalmic eye ointment. 2P imaging was performed at a wavelength of 930 nm. The mouse was imaged for 2 h after which it recovered in less than 10 min.

Light for 1P imaging *in vivo* was patterned via a digital micromirror device to selectively illuminate the targeted cell body, as described above.

### 2.6 Data Analysis

All analysis was performed in MATLAB. Cell fluorescence was measured from the regions of interest (ROIs) manually selected to lie on the cell membrane. 1P and 2P recordings from the same cell used identical analysis ROIs. Fluorescence from cell-adjacent illuminated areas was used to estimate the background.

To estimate shot noise for an ideal detector (as an upper bound of performance), we converted camera counts to incident photons by dividing by the camera quantum efficiency (QE=∼67% at 525 nm) and multiplying by the conversion factor (CF=0.46 photoelectrons/digital count).

## 3 Results

### 3.1 Shot Noise Constrains Functional Fluorescence Imaging

Shot noise imposes a fundamental limit on imaging performance. A source that generates, on average, N-detected photons will have fluctuations with standard deviation $\sqrt{N}$. To detect a 1-ms spike (ΔF/F∼10%) with an SNR of 10, it requires determining fluorescence to 1% precision in 1 ms. We adopt 1% precision in 1 ms as a reasonable standard for a high-SNR voltage recording. Due to shot noise, this standard requires detecting at least $10^{4}\;\text{photons}/\mathrm{ms}$ or $10^{7}\;\text{photons}/s$.

More generally, imaging an event of magnitude ΔF/F=β with a given SNR requires determining fluorescence to a precision of β/SNR. The photon flux (Γ) required for a measurement rate (f), signal level (β), and SNR is

$$\Gamma =f\cdot {\left(\frac{\mathrm{SNR}}{\beta }\right)}^{2}.$$ (1)

If one wishes only to detect spikes in pyramidal cells, one might tolerate SNR=3 and f=400  Hz. A highly sensitive GEVI could give β=0.2 for a spike.^6^ Under these conditions, the minimum detection rate is $\Gamma =9\times 10^{4}\;\text{photons}/s$. In comparison, for a typical calcium imaging scenario, SNR=10, β=1, and f=30  Hz,^30^ implying Γ=3000  photons/s, which is 30-fold less than even low-SNR spike detection via voltage imaging. Thus, the brief duration of voltage spikes and the low fractional sensitivity of existing voltage indicators conspire to make voltage imaging a very photon-greedy technique.

Filtering in space or time can increase the effective value of N at a given pixel, at the cost of lower spatial or temporal resolution, but filtering does not change the $\sqrt{N}$ shot noise scaling. We discuss advanced analysis techniques below.

### 3.2 1P Versus 2P Excitation of Commonly Used Voltage Indicators

**Fig. 1:**
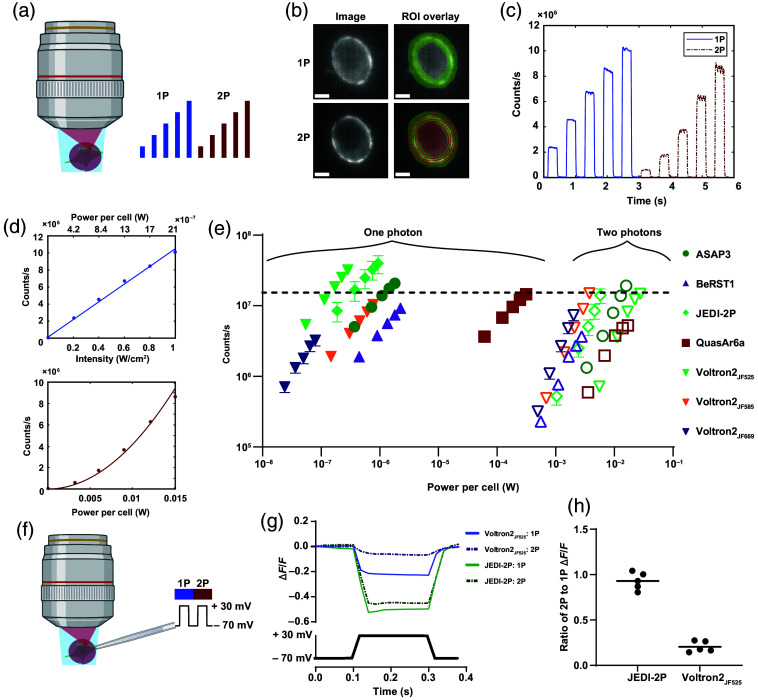
Comparison of 1P and 2P brightness and sensitivity of fluorescent voltage indicators. (a) Diagram of the experiment. HEK cells were sequentially illuminated with wide-field 1P light in steps of increasing intensity and then by spiral scan 2P steps of increasing intensity. (b) Example HEK cell expressing the GEVI ASAP3. The 2P spiral scan pattern is shown in red, and the analysis ROI is shown in green. Scale bars=5  μm. (c) Example single-trial data for a cell expressing ASAP3. (d) Top: fluorescence in panel (c) as a function of 1P intensity with a linear fit. Bottom: fluorescence as a function of 2P power with quadratic fit. (e) Log–log plot of count rate versus optical power on the cell for seven voltage indicators. 1P data, filled symbols; 2P data, empty symbols. Error bars are standard error of the mean from at least n=8 cells. The excitation wavelengths used for 1P (2P) excitation of each of the reporters were ASAP3 488 nm (930 nm), BeRST1 635 nm (850 nm), JEDI-2P 488 nm (930 nm), QuasAr6a 635 nm (900 nm), ${\text{Voltron}2}_{525}$ 488 nm (930 nm), ${\text{Voltron}2}_{585}$ 594 nm (1100 nm), and ${\text{Voltron}2}_{669}$ 635 nm (1220 nm). A horizontal line is shown at $1.5\times 10^{7}\;\text{counts}/s$, equivalent on our camera to $10^{7}$ impinging photons/s. (f) Whole-cell patch clamp protocol for measuring voltage sensitivity under 1P and 2P excitation. (g) Average voltage responses of JEDI-2P and ${\text{Voltron}2}_{525}$ under 1P and 2P illumination. (h) Ratio of voltage contrast under 2P versus 1P illumination for JEDI-2P and ${\text{Voltron}2}_{525}$, n=5 cells per construct.

We compared the brightness of commonly used voltage indicators in HEK293T (HEK) cells under alternating wide-field 1P illumination and 2P spiral illumination with an 80-MHz pulsed laser. Point-scanning 2P imaging typically uses a single-element detector such as a photomultiplier tube. We used a shared camera-based detection path for both 1P and 2P imaging to ensure equal photon detection efficiencies and to facilitate quantitative comparisons [Figs. 1(a)–1(c); Sec. 2: Methods]. For real-world 2P imaging, the choice of the detector can impact system performance via detector electronic noise and quantum efficiency. In our measurements, the per-pixel count rates were high enough that electronic noise contributed little to overall noise, compared with shot noise. To facilitate the translation of our results to other systems, we converted digital camera counts to photons incident on the camera using the known camera quantum efficiency and digitizer gain. This provides a “best-case” signal estimate for an ideal detector.

We compared a voltage-sensitive dye (BeRST1^31^), an opsin-derived GEVI imaged via intrinsic retinal fluorescence (QuasAr6a^32^), a chemigenetic FRET-opsin GEVI (Voltron2^33^), and two GEVIs that couple a voltage-sensitive phosphatase (VSP) to a circularly permuted partner fluorophore (ASAP3^4^ and JEDI-2P^6^).

As expected, the fluorescence scaled linearly with 1P illumination intensity and quadratically with 2P intensity [Fig. 1(d)]. For all but one indicator, 2P illumination required at least $10^{4}$-fold greater time-averaged power per cell to achieve comparable counts to 1P illumination [Fig. 1(e)]. The ratio of 2P-to-1P powers for QuasAr6a was only ∼300 due to the requirement for high-intensity 1P illumination and selection rules that favor 2P over 1P excitation in opsins.^34^ Consistent with prior reports,^1^ we found that chemigenetic indicators were brighter under both 1P and 2P illumination than their purely protein-based counterparts, though our measurements did not assign the relative contributions of expression level versus per-molecule brightness.

The huge difference in optical power requirement between 1P and 2P (80 MHz) excitation is consistent with published reports: 2P imaging of JEDI-2P was reported at a power of 9 to 12  mW/cell,^6^ whereas 1P imaging of similar GFP-based GEVIs is typically performed at 1 to $10\;W/{\mathrm{cm}}^{2}$,^4^^,^^6^ corresponding to 1 to 10  μW/cell. Estimates based on tabulated 1P and 2P absorption coefficients^35^ give a similar factor of $∼10^{4}$ difference in power efficiency (see the Supplementary Material for calculation).

We then measured the voltage sensitivity of one representative from each GEVI family, comparing 1P and 2P illumination. Using whole-cell voltage clamp in HEK cells [Fig. 1(f)], we found that the contrast (ΔF/F per 100 mV) of the VSP-based JEDI-2P was similar for 1P and 2P illumination. The opsin-based chemigenetic indicator ${\text{Voltron}2}_{525}$ showed voltage sensitivity under 1P but not 2P illumination [Figs. 1(g) and 1(h)]. Loss of voltage sensitivity under 2P illumination of FRET-Opsin GEVIs has been reported previously.^36^ We recently determined the photophysical mechanism underlying this effect^37^ but did not pursue 2P imaging of FRET-Opsin GEVIs here.

### 3.3 Testing the Dependence of 1P and 2P Signals as a Function of Depth

**Fig. 2:**
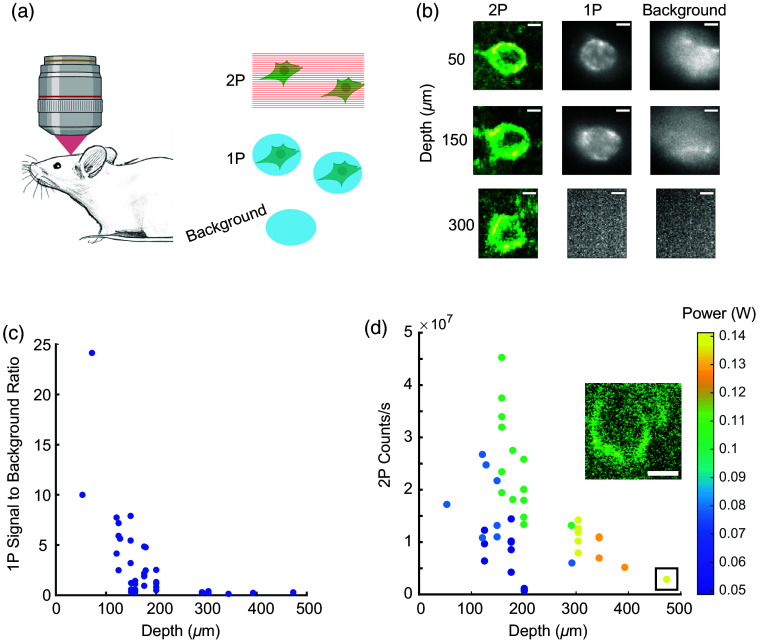
Depth-dependent 1P and 2P signals in the brain. (a) Experimental protocol. In a mouse expressing JEDI-2P, raster-scanned 2P (λ=920  nm) and DMD-patterned 1P imaging (λ=488  nm) were alternately applied to neurons at different depths. 1P illumination patterned to cell-free regions was used to estimate the background signal. (b) Example 1P and 2P images of cells at three depths. Scale bars=5  μm. (c) Estimated signal-to-background ratio for n=43 neurons under patterned 1P illumination. (d) Mean count rate from the membranes of n=43 neurons under 2P illumination. The color indicates excitation power. The inset shows the cell at 473  μm depth (boxed on the graph). Scale bar=5  μm.

To characterize the depth dependence of 1P and 2P voltage imaging in brain tissue, we sparsely expressed soma-localized JEDI-2P in the mouse cortex. Chien et al.^38^ previously showed that for voltage imaging in brain tissue, restricting 1P illumination to the soma led to an approximately eightfold improvement in signal-to-background ratio compared with wide-field illumination, by minimizing the background from off-target illumination. We thus compared the soma-targeted 1P imaging and raster-scanned 2P imaging at several depths [Figs. 2(a) and 2(b); Sec. 2: Methods].

With 1P illumination, JEDI-2P-expressing cells were resolvable down to d∼200  μm. The challenge for 1P imaging at greater depths was not shot noise but rather a decrease in signal-to-background ratio. At depths >200  μm, cells were not distinguishable from the background by 1P imaging with patterned 488 nm excitation [Fig. 2(c)].

With 2P illumination, cells were resolvable to d=473  μm, the greatest depth we tested [Fig. 2(b)]. As the cell depth increased, we increased the 2P laser power to maintain a sufficient count rate to resolve the cells, up to $P_{0}=140\;\mathrm{mW}$ at d=473  μm.

### 3.4 Thermal Limits to 2P Excitation Power in Brain Tissue

The heating caused by 2P illumination can transiently perturb neural function and at high levels can damage tissue. Most ion channel gating properties have a $Q_{10}$ (i.e., ratio of rates at temperatures separated by 10°C) between 1 and 3.^39^ Changes of 1°C can cause changes in neuronal firing rates.^40^ In rodents, brain temperature may fluctuate under physiological conditions by up to 4°C,^41^ and implantation of a glass imaging window may lead to some local brain cooling, partially canceling the effect of laser heating. Podgorski and Ranganathan^42^ found lasting damage after continuous illumination of a $1\;{\mathrm{mm}}^{2}$ scan at 250 mW, corresponding to a steady-state temperature change of ∼5°C.

The relation between laser power and heating depends on the scan area, scan pattern, and measurement duty cycle. For a $1\text{-}{\mathrm{mm}}^{2}$ square scan pattern, Podgorski and Ranganathan found steady-state temperature coefficients between 0.012 and 0.02°C/mW at wavelengths from 800 to 1040 nm, equivalent to a temperature rise of <2°C at 100 mW illumination. They simulated the dependence of temperature rise on the scan area and found a weak dependence. Their results predict a maximum temperature coefficient of 0.03°C/mW for a square scan of side length 20  μm (representing a single neuronal soma). The precise value of this coefficient depends both on the brain region and the wavelength.^43^ Finally, Podgorski and Ranganathan showed that reducing the illumination duty cycle to 10 s on and 20 s off allowed brighter illumination to be used during “on” periods while still keeping time-averaged heating beneath the damage threshold. Some experiments may permit low-duty cycle imaging, whereas others may not. Hereafter, we use 200 mW as a reasonable upper bound on the power, acknowledging that this limit may be up to approximately twofold higher (or lower) depending on many experimental details.

### 3.5 Estimating Measurable Cells as a Function of Depth

Shot noise places an upper bound, $N_{\text{cells}}^{2P}$, on the number of cells that can be measured simultaneously via 2P illumination with a given illumination power and SNR. Under a protocol that sequentially visits single cells, $N_{\text{cells}}^{2P}$ depends on both the brightness and voltage sensitivity (see the Supplementary Material for derivation),

$$N_{\text{cells}}^{2P}=\frac{A\tau P^{2}\beta^{2}\phi }{{\mathrm{SNR}}^{2}}.$$ (2)

Here, A is the brightness coefficient derived from the HEK cell experiments [Eq. (S1) in the Supplementary Material], τ is the integration time, P is the laser power at the focus, β=ΔF/F per spike, ϕ is the fraction of the scan that intersects with the cell membrane (i.e., the imaging duty cycle), and SNR is the target ratio of the spike amplitude to shot noise. The value of A is specific to the laser repetition rate, pulse width, focal parameters, and detection optics. We discuss the effects of varying these parameters below. For an analysis that includes the effect of light scattering on depth-dependent collection efficiency, see Ref. 15.

The parameter ϕ approaches 1 for perfectly membrane-targeted illumination. To estimate ϕ for a raster scan over a bounding box around a single cell body, we examined 2P images of pyramidal cells with membrane-targeted fluorescent tags in cortex layer 2/3. In these images, the membrane-labeled area fraction within the bounding box was $\phi_{\mathrm{bb}}=0.18\pm 0.07$ (mean ± std, n=10 cells). For a raster scan over multiple sparsely expressing cells, ϕ is lower than $\phi_{\mathrm{bb}}$ by a factor of the sparsity. The low values of ϕ for raster-scanned 2P imaging are a consequence of the membrane-localized signal and highlight the importance of membrane-targeted illumination. However, precise targeting of the illumination to the membrane increases sensitivity to motion artifacts. The ULoVE technique brackets the membrane with pairs of spots and thereby mitigates the effect of small motions, at the cost of less-than-optimal membrane targeting of the spots.^4^

**Fig. 3:**
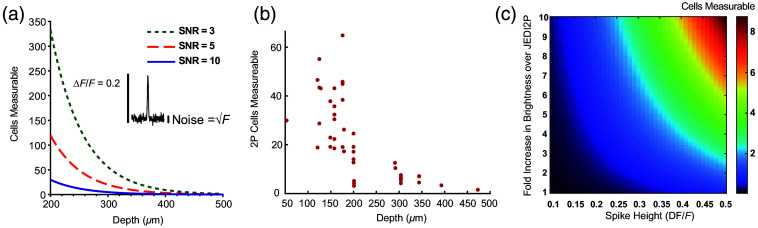
Scaling of 2P voltage measurements with depth and with GEVI properties. (a) Predicted number of simultaneously measurable cells as a function of depth, based on brightness derived from HEK cells expressing JEDI-2P (Fig. 1). We assumed a spike contrast of β=0.2; target SNRs of 3 (green dotted), 5 (blue dashed), or 10 (red solid) in an integration time τ=1  ms; a total power $P_{0}=200\;\mathrm{mW}$, targeting fraction ϕ=1, scattering length $l_{e}=112\;\mu m$,^44^ and a detector with perfect quantum efficiency. (b) Predicted number of simultaneously measurable cells at SNR=10 and 200 mW power for each experimentally measured single-cell count rate reported in Fig. 2(d). (c) Number of simultaneously measurable cells under 2P illumination at a depth of 500  μm, assuming brightness and contrast improvements of future GEVIs, target SNR of 10, and all other parameters as in panel (a).

Equation (2) can be used to predict the scaling of $N_{\text{cells}}^{2P}$ as a function of depth, d, for 2P voltage imaging. The power at a laser focus decays exponentially with d, with an extinction length, $l_{e}$, in brain tissue. At λ=920  nm, $l_{e}$ is between 112 (Refs. 15 and 44) and 155  μm.^45^ Due to the quadratic dependence of the 2P signal on focal intensity, the 2P signal decays with a length constant of $l_{e}/2$. Substituting $P=P_{0}e^{-d/l_{e}}$ into Eq. (2) implies a decay in $N_{\text{cells}}^{2P}$ by a factor of 10 for every 130 to 180  μm increase in d [Fig. 3(a)], assuming constant power $P_{0}$ into the tissue.

We applied Eq. (2) to the instantaneous count rates measured from the cell membranes [Fig. 2(d)] to estimate $N_{\text{cells}}^{2P}$ for ϕ∼1, i.e., a perfectly membrane-targeted annular scan pattern. We converted the digital camera counts to collected photon rates to provide an upper performance bound for a perfect detector. Modern back-illuminated sCMOS cameras have detection efficiencies that approach 100%. We assumed β=0.2, based on the reported spike response of JEDI-2P^6^ and a target shot noise-limited SNR of 10 in a 1 kHz bandwidth, and extrapolated the count rates to an input power of $P_{0}=200\;\mathrm{mW}$. The estimated number of measurable cells decreased quickly at d>200  μm and dropped below three at d>470  μm and input power 200 mW [Fig. 3(b)]. These results are similar to the prediction from the simple model using the 2P count rates from our HEK cell experiments [Fig. 3(a), blue line].

The palette of available voltage indicators is continually improving.^46^ We therefore considered the scaling of $N_{\text{cells}}^{2P}$ with changes in brightness and spike height (β). $N_{\text{cells}}^{2P}$ depends linearly on A and quadratically on β [Fig. 3(c)]. An order-of-magnitude increase in molecular brightness coupled with a 2.5-fold increase in β compared with JEDI-2P could enable high SNR measurement of >8 cells at depths up to 500  μm using the optical configuration we considered above.

### 3.6 Effect of GEVI Kinetics

Some GEVIs have response times that are slow compared with the duration of a spike. On the one hand, this blunts the amplitude of the spike response; on the other hand, it permits one to average for longer to detect whether a spike has occurred (assuming that the interval between spikes remains long compared with the recovery time of the GEVI). Here, we analyze this tradeoff.

**Fig. 4:**
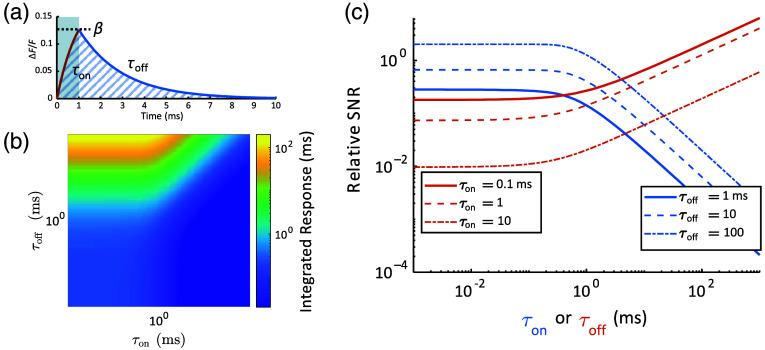
Effect of reporter kinetics on signal. (a) Response of a reporter to a 1-ms voltage pulse. An exponential rise with time constant $\tau_{\text{on}}$ reaches a maximum of β followed by an exponential decay with time constant $\tau_{\text{off}}$. The signal comprises the total area under both response phases (cross-hatched). (b) Area under the curve in panel (a) as a function of $\tau_{\text{on}}$ and $\tau_{\text{off}}$, keeping constant pulse duration (1 ms) and steady-state voltage sensitivity ($\beta_{\mathrm{ss}}=0.2$). Increasing $\tau_{\text{off}}$ allows longer integration time, whereas increasing $\tau_{\text{on}}$ truncates the response. (c) Effects on SNR of changing $\tau_{\text{on}}$ or $\tau_{\text{off}}$ while keeping the other fixed. The fixed parameter is shown in the legend, and variable 1 is indicated by the x-axis. At large $\tau_{\text{on}}$, $\mathrm{SNR}∼1/\tau_{\text{on}}$. At large $\tau_{\text{off}}$, $\mathrm{SNR}∼\tau_{\text{off}}^{1/2}$.

Consider a GEVI subjected to a voltage step that induces a steady-state change in fluorescence, $\frac{\Delta F}{F}=M$. Assume that the GEVI responds to a voltage step of duration t with exponential response time constants $\tau_{\text{on}}$ and $\tau_{\text{off}}$ [Figs. 4(a) and 4(b)]. We define R as the area under the curve of ΔF/F versus t (see the Supplementary Material for derivation)

$$R=M(t+(\tau_{\text{off}}-\tau_{\text{on}})(1-e^{-t/\tau_{\text{on}}})).$$ (3)

Equation (3) assumes the collection of the entire tail of the decay and thus provides an upper bound to the signal. For a GEVI with a symmetric upstroke and downstroke ($\tau_{\text{on}}=\tau_{\text{off}}$), R=M t. For a finite integration time equal to $t+\tau_{\text{off}}$, the relation of SNR and R is (see the Supplementary Material for derivation)

$$\mathrm{SNR}=\frac{\mathrm{RF}\phi }{\sqrt{F\phi (t+\tau_{\text{off}})}}.$$ (4)

A large $\tau_{\text{on}}$ will decrease the magnitude of the response β to a short electrical spike, decreasing instantaneous SNR proportionally. A large $\tau_{\text{off}}$, however, increases the duration of the impulse response, increasing the duration of the signal that can be integrated and leading to an increase in SNR proportional to $\tau_{\text{off}}^{1/2}$ [Eq. (S7) in the Supplementary Material, Fig. 4(c)]. When $\tau_{\text{on}}$ and $\tau_{\text{off}}$ can be independently chosen, $\tau_{\text{on}}$ should be minimized (with diminished effect once $\tau_{\text{on}}$ is below the spike width) and $\tau_{\text{off}}$ maximized (while remaining short compared with the interspike interval). Often, these time constants are biophysically related. If $\tau_{\text{on}}\approx \tau_{\text{off}}=\tau$, then $\mathrm{SNR}∼\tau^{-1/2}$ [see Eq. (S7) in the Supplementary Material]. That is, faster GEVIs are better than slower ones in terms of shot noise-limited SNR, all else being equal.

### 3.7 Effect of Optical Parameters on 2P Fluorescence

Advances in 2P voltage imaging typically have two aims: (1) increasing the number of cells, N, which are sampled with a high enough revisit rate to capture all spikes and (2) increasing fluorescence per cell to improve the shot noise-limited SNR. In many cases, these two aims are in tension.

#### 3.7.1 Changing numerical aperture

**Fig. 5:**
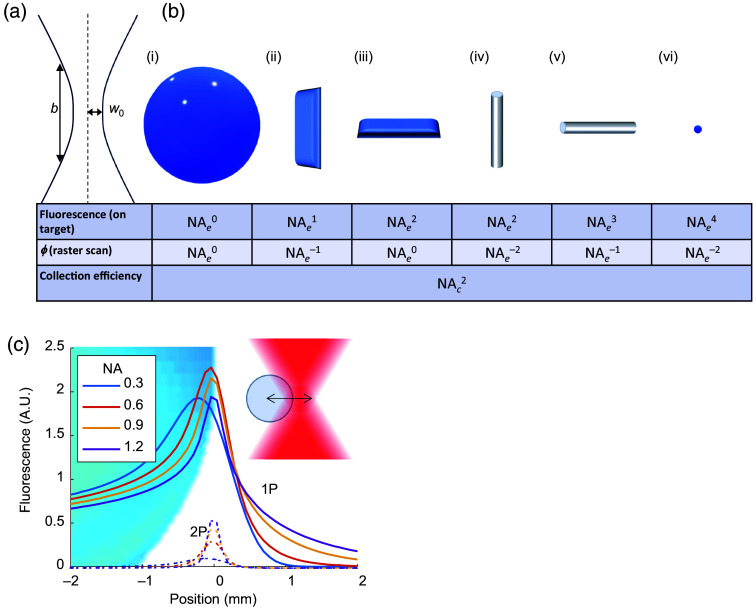
Scaling of fluorescence with numerical aperture, sample geometry, and excitation modality. (a) Geometry of a Gaussian beam, showing the width ($W_{0}∼1/\mathrm{NA}$) and waist ($b∼{1/\mathrm{NA}}^{2}$). (b) Scaling of total 2P fluorescence as a function of excitation ${\mathrm{NA}}_{e}$ for different sample geometries. All slender dimensions are assumed to be $\ll W_{0}$, and all extended dimensions are assumed to be ≫b. In a planar raster scan, the fraction of time that a subwavelength structure is excited, ϕ, depends on the focus width and hence the ${\mathrm{NA}}_{e}$. In cases (iii), (v), and (vi), we assume that the object is perfectly in focus, i.e., in the axial plane where focus size is minimum. The collection efficiency for all geometries depends on the collection solid angle $∼NA_{c}^{2}$. To calculate the total signal for targeted illumination, multiply the first and third lines; for a raster scan, multiply all three lines. (c) Total fluorescence evoked by the intersection of a laser focus and a spherical membrane, 10  μm diameter. We compared 1P and 2P excitations with equal ${\mathrm{NA}}_{c}$ and ${\mathrm{NA}}_{e}$, with powers adjusted to match per-molecule excitation rates at the focus at NA = 1.2. The much smaller 2P focal volume led to a 5.3-fold smaller maximum fluorescence at the highest NA (and even greater discrepancy at lower NA) and a 3-fold greater sensitivity to misalignment compared with 1P excitation.

We distinguish between the numerical aperture of excitation (${\mathrm{NA}}_{e}$) and of collection (${\mathrm{NA}}_{c}$). Often, ${\mathrm{NA}}_{c}$ is set by the objective NA, whereas ${\mathrm{NA}}_{e}$ may be lower as a result of underfilling the objective back aperture. The photon detection efficiency (PDE) scales as $\mathrm{PDE}∼{\mathrm{NA}}_{c}^{2}$. The effective ${\mathrm{NA}}_{c}$ may be increased beyond the objective NA by collecting high-angle fluorescence photons with either reflective^47^ or fiber-optic^48^ auxiliary collectors. The effect of ${\mathrm{NA}}_{e}$ on the 2P signal depends on the sample geometry. Within the Gaussian beam approximation [Fig. 5(a)], the width of the focus scales as

$$w_{0}\propto \frac{1}{{\mathrm{NA}}_{e}}.$$

The intensity, I, at the focus scales inversely with the cross-sectional area (so $I\propto {\mathrm{NA}}_{e}^{2}$), and the rate of 2P excitation per molecule, E, scales with the intensity squared. Hence,

$$E\propto {\mathrm{NA}}_{e}^{4}.$$

The axial extent of the beam waist scales as

$$b\propto \frac{1}{{\mathrm{NA}}_{e}^{2}}.$$

The total signal from a volume element depends on the number of fluorophores excited. In a three-dimensional bulk solution—an approximation of 2P ${\mathrm{Ca}}^{2+}$ imaging when the beam waist is significantly smaller than a single cell—the volume scales approximately as $V∼w_{0}^{2}b\propto \frac{1}{{\mathrm{NA}}_{e}^{4}}$. The total fluorescence emission $\Gamma_{2P}$ scales as V×E. Hence, in bulk solution, $\Gamma_{2P}\propto {\mathrm{NA}}_{e}^{0}$, so that the total collected fluorescence, F, scales only with ${\mathrm{NA}}_{c}$ as $F∼{\mathrm{NA}}_{c}^{2}$.

In contrast, for 2P voltage imaging, the fluorescence rate from the sample scales with ${\mathrm{NA}}_{e}$ raised to a power between 1 and 3, depending on the orientation and geometry of the membranes in the focal spot [Fig. 5(b), first row]. This different scaling arises because the fluorophores are arranged in a 2D membrane instead of a 3D volume. The scaling of membrane excitation argues strongly for maximizing the ${\mathrm{NA}}_{e}$ for 2P voltage imaging. On the other hand, a smaller excitation spot leads to (a) a higher rate of photobleaching and possibly photodamage and (b) greater sensitivity to misalignment between the focus and the sample, e.g., from sample motion [Fig. 5(c)]. The optimal ${\mathrm{NA}}_{e}$ for 2P voltage imaging *in vivo* likely involves a sample-dependent balance of these considerations.

#### 3.7.2 Advanced scanning modalities

State-of-the-art techniques for high-speed 2P microscopy often involve shaping and splitting the excitation in space and/or time.^4^^,^^5^^,^^20^^,^^49^^,^^50^ Some 2P imaging modalities use beam splitters to split each laser shot into a series of pulses, which arrive sequentially at different locations in the sample.^17^^,^^20^^,^^23^^,^^49^ Other modalities expand the laser beam to cover more than a single diffraction-limited spot (e.g., multifocal,^4^^,^^16^^,^^23^^,^^50^^,^^51^ temporal focusing,^5^^,^^50^^,^^52^ or Bessel beam^53^ microscopies). For a review of recent advances in high-speed 2P microscopy, see Ref. 54. Here, we discuss the effects on optical SNR of beam splitting and shaping. First, we consider the case where illumination is limited by total power into the sample (e.g., by heating). Then, we consider the implications of limits on peak energy density at the laser focus (e.g., photodamage and saturated excitation).

Consider the limiting case of a single diffraction-limited point focus, targeting a single cell, giving a fluorescence count rate of Γ. If one now wishes to image two cells, one could adjust the scan pattern to alternate between the cells, targeting each with a 50% duty cycle. On account of the duty cycle, each cell gives a count rate of Γ/2. Alternatively, one could split the spot in two and image each cell with 100% duty cycle but 50% of the power. On account of the $P^{2}$ dependence of 2P fluorescence, the time–average count rate per cell becomes Γ/4. Thus, from a shot noise perspective, the alternating strategy is better than pulse splitting. The third approach is to split the focal spot in two and cut the laser repetition rate in half while maintaining constant time–average power into the sample (i.e., doubling the input pulse energy). Then, the time–average count rate per cell is back up to Γ/2.

More generally, consider a laser with repetition rate f that is split into N diffraction-limited spots while maintaining constant total power in the sample. This scenario applies to pulse-splitting techniques and also to temporal focusing, where a single laser focus is expanded into a pancake-shaped excitation volume that covers many diffraction-limited spots. The excitation rate per diffraction-limited spot is proportional to $1/N^{2}$. If the additional spots are used to increase the number of cells targeted, then the fluorescence count rate per cell also scales as $1/N^{2}$, and the SNR per cell scales as 1/N. On the other hand, if the additional spots are used to sample more densely from a fixed number of cells, the fluorescence count rate per cell scales as 1/N, and the SNR per cell scales as $1/\sqrt{N}$. If the laser repetition rate is varied while maintaining constant power and arrangement of spots, the mean count rate per cell scales as 1/f. Thus, minimizing N and minimizing f (i.e., using a single-point focus at a low repetition rate) maximize the signal per cell, provided that the focal energy density remains below saturation and that the target voxel rate can be achieved.

This scaling is consistent with the recent report of Sims et al.^5^ that decreasing the laser repetition rate 320-fold from 80 MHz to 250 kHz while keeping power constant increased the number of cells imaged at constant SNR from 1 cell/125 mW to 17 cells/125 mW. Similarly, increasing the spot area by a factor of ∼100 to cover an entire cell implies a 10-fold drop in $N_{\text{cells}}$ compared with our point-scanning estimates [Fig. 3(a)], predicting fewer than ∼30 cells measurable with an 80-MHz laser with SNR of 10 at the brain surface. Sims et al.^5^ also found that a whole-cell temporally focused scanless system performed better with speckled illumination that approximated a point array than with more homogeneous illumination, supporting the view that sparser excitation increases fluorescence count rate and hence shot noise–limited SNR.

Considering the above analysis, why is pulse splitting sometimes advantageous? In some ${\mathrm{Ca}}^{2+}$ or glutamate imaging experiments, the goal is to record from as many sources as possible. Due to the availability of high-contrast (i.e., large β) ${\mathrm{Ca}}^{2+}$ and glutamate indicators, the shot noise–limited SNR achievable from a single diffraction-limited focus can be far higher than needed. In this case, splitting up the laser focus can increase the voxel sample rate, at an acceptable tradeoff in SNR. Ultimately, different experiments may favor different tradeoffs between SNR and voxel sample rate. For voltage imaging, SNR is usually the priority, whereas, for other modalities, voxel rate may be more important.

#### 3.7.3 Effect of nonlinear saturation

**Fig. 6:**
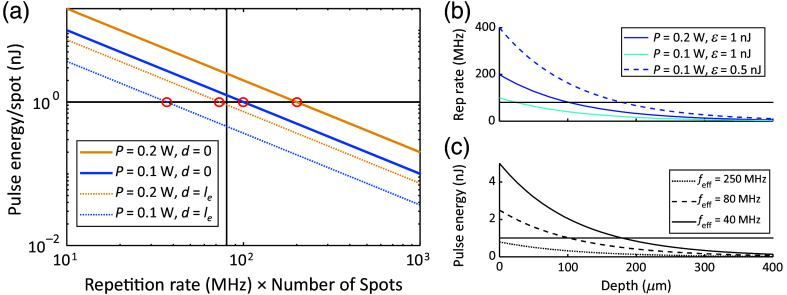
Optimal temporal and spatial splitting. (a) At fixed total power, the per-spot pulse energy is inversely proportional to the effective repetition rate, $f_{\mathrm{eff}}=f\times N_{\text{spots}}$. SNR is increased by increasing focal pulse energy up to the threshold (1-nJ horizontal line shown). Therefore, the optimal effective repetition rate lies at the intersection of the iso-power line with the threshold (circled in red). At a nonzero depth (dotted lines, $l_{e}$ = attenuation length), a lower repetition rate is needed to produce the same focal pulse energy. (b) For a single diffraction-limited focal spot, a total power of 200 mW, and a pulse energy threshold of 1 nJ, the optimal $f_{\mathrm{eff}}$ goes beneath 80 MHz (horizonal line) at ∼100  μm depth. Decreasing the threshold focal energy (e.g., to 0.5 nJ) increases the optimal repetition rate at a given depth. (c) For a fixed $f_{\mathrm{eff}}$, the focal pulse energy decays exponentially with depth. At $f_{\mathrm{eff}}=250\;\mathrm{MHz}$, a 1-nJ pulse is not achievable at any depth. At $f_{\mathrm{eff}}=40\;\mathrm{MHz}$, the pulse energy crosses the 1 nJ threshold at ∼200  μm depth.

Maximizing focal intensity is only beneficial up to a point. Saturation of the 2P excitation typically occurs at diffraction-limited pulse energies of ∼1  nJ^14^ at the focus, and nonlinear local photodamage may occur at similar pulse energies.^55^^–^^57^ For a fixed pulse duration and spot size, this limit is independent of f. We define the effective repetition rate, $f_{\mathrm{eff}}=f\times N_{\text{spots}}$. The optimal $f_{\mathrm{eff}}$ is the repetition rate at which the focal pulse energy density reaches but does not exceed the saturation or damage limit [Fig. 6(a)]. However, if emitted photons are collected in a single-element detector (as opposed to a camera), then the interval between laser pulses should also be several-fold larger than the electronic excited state lifetime (typically 2 to 4 ns) to avoid crosstalk between successive pulses. This constraint limits the pulse rate in the sample to <∼150  MHz.

Simultaneously approaching the 2P saturation energy at the focus (∼1  nJ) and the maximum power into the sample (∼200  mW) implies a depth-dependent optimum value of $f_{\mathrm{eff}}$ [Fig. 6(b)]. For example, for an 80 MHz laser illuminating the brain with 200 mW, a single focal spot will exceed the 1 nJ limit at the brain surface. Splitting the focus into three diffraction-limited spots ($f_{\mathrm{eff}}=240\;\mathrm{MHz}$) allows each spot to remain beneath the 1-nJ limit while using the full 200-mW budget, though time-resolved fluorescence detection at this frequency would induce crosstalk between successive pulses. Meanwhile, at a depth of 5 $l_{e}$, where $l_{e}$ is the exponential attenuation length, optimal $f_{\mathrm{eff}}\approx 1$ to 10 MHz.^14^ A 2.5 MHz fiber-based soliton laser gave a 26-fold greater average signal than an 80 MHz Ti-to-Sa laser of equal time–average power at depths up to 680  μm in the live mouse brain.^14^ In summary, the $f_{\mathrm{eff}}$ should be tuned to the power limit, focal energy threshold, and imaging depth for each experiment.

The effective repetition rate must also be high enough to visit each measurement point at least once per measurement cycle. For example, an 800 kHz laser could visit 800 points at a 1 kHz revisit rate (assuming a suitable scanner existed) and would provide 100-fold higher time–average count rate (and 10-fold higher shot noise–limited SNR) than the same laser power delivered at 80 MHz, assuming a subsaturation pulse energy. Mechanical and acousto-optical scanners have finite slew rates, which can limit the number of cells measurable within 1 ms. When this limit is below the limit set by optical SNR, beam splitting may increase the number of measurable cells.

#### 3.7.4 Optimizing the Polarization

For membrane-localized chromophores, the signal can be increased by aligning the excitation polarization with the transition dipole of the chromophore.^58^ For 1P excitation, this effect scales as $\cos^{2}\;\theta$, where θ is the angle between the excitation polarization and the transition dipole, and the average is taken over the distribution of molecular orientations. The magnitude of the polarization-dependent effect is characterized by $(\Delta F/\overset{¯}{F}{)}_{\mathrm{pol}}$, where $\overset{¯}{F}$ is the fluorescence for unpolarized excitation and $\Delta F=F_{\max}-\overset{¯}{F}$, where $F_{\max}$ is the fluorescence for optimal polarization. At the cell periphery, where the optic axis lies in the plane of the membrane, this effect magnitude was $(\Delta F/\overset{¯}{F}{)}_{\mathrm{pol}}=54\%$ for the dye BeRST1, 20% for ASAP1, 13% for QuasAr3, 12% for ArcLight, and 4.5% for the FRET-opsin GEVI mNeon-Ace.^58^

For 2P excitation, polarization sensitivity scales as $\left\langle \cos^{4}\;\theta \right\rangle$^59^ and can lead to several-fold polarization-dependent changes in fluorescence from neurites with membrane-bound reporters.^60^ Thus, 2P voltage imaging systems could improve their power efficiency substantially by ensuring linearly polarized excitation at the sample and selectively targeting cell membranes that have a favorable orientation relative to the laser polarization or by modulating polarization during a scan to match the orientation of the target membranes.

### 3.8 Can Advanced Analysis Techniques Overcome the Shot Noise Limit?

Consider the goal of detecting whether a spike occurred (hypothesis $H^{\left(1\right)}$) or did not occur ($H^{\left(0\right)}$) during a measurement time τ. The mean number of detected photons in the case of a spike is $n_{1}$, and that in the absence of a spike is $n_{0}$. The probability distributions for the number of detected photons in the two cases are then given by Poisson distributions with means $n_{1}$ and $n_{0}$ respectively

$$p(n|H^{\left(1\right)})=\text{Poisson}(n;n_{1}),$$

and

$$p(n|H^{\left(0\right)})=\text{Poisson}(n;n_{0}).$$

If the number of photons collected in either scenario is not large and the contrast $\beta =(n_{1}-n_{0})/n_{0}$ is also small, then the two probability distributions overlap: a given set of detected photons could have been produced by either the presence or absence of a spike. In such cases, no analysis algorithm can unambiguously determine whether a spike occurred; at best, one can determine the relative probabilities of the two hypotheses. This argument is analyzed in detail in Ref. 12.

Voltage signals corresponding to spikes are typically correlated across multiple pixels and sometimes across frames (depending on the frame rate and spike duration). As the photon shot noise is statistically independent among all pairs of pixels, the relative contribution of shot noise can be diminished by weighted averaging across pixels and possibly across frames. If the expected number of photon detections at pixel i is $\left\langle n_{i}\right\rangle$ and a filter assigns weight $a_{i}$ to the pixel, then the expected signal is $S=\underset{i}{\sum }a_{i}\langle n_{i}\rangle$, and the variance in this quantity due to photon shot noise is $\sigma_{S}^{2}=\underset{i}{\sum }a_{i}^{2}\langle n_{i}\rangle$. The art of voltage imaging analysis comprises determining the $a_{i}$ to maximize the difference between $p(S|H^{\left(1\right)})$ and $p(S|H^{\left(0\right)})$.

Determination of the weights $a_{i}$ can be via simple manual or activity-based selections of regions of interest or via optimal detection algorithms, e.g., as in Ref. 61. When signals from multiple sources overlap, a variety of unmixing algorithms are useful.^62^^–^^66^ It is possible even to apply a filter during image acquisition to reduce the data burden.^67^ Recently introduced machine learning algorithms^24^^,^^25^ can help determine the optimal weighting of pixel signals. Noise reduction and signal extraction algorithms can play an important role in voltage imaging data analysis.

None of these techniques, however, overcomes the fundamental uncertainty that different voltages can give identical photon distributions at the detector. It is straightforward to simulate voltage imaging datasets with realistic shot noise (as well as other noise sources), where ground truth is known. Analysis methods should be validated against simulated data, and false-positive and false-negative spike detection rates should be quantified. Claims that denoising methods can “overcome fundamental limits”^23^ of shot noise are misleading.

## 4 Conclusions

Equation (2) places severe constraints on the number of neurons that will be measurable at d>300  μm with 2P voltage imaging, even with substantial improvements in GEVI brightness and voltage sensitivity [Fig. 3(c)]. These findings indicate that 2P imaging of hundreds of neurons with high SNR at depth >300  μm will require an order-of-magnitude improvement in 2P GEVIs or qualitatively new approaches to imaging. Given the current state of the art, one can maximize SNR and number of measurable cells at depth using excitation with high numerical aperture, low repetition rate (1 to 10 MHz), short pulses (<100  fs), optimized polarization, and membrane-targeted illumination with real-time compensation for tissue motion. Optimal imaging can be achieved by customizing spatial multiplexing, repetition rate, and/or excitation NA for the target imaging depth and power limit. Experiments that allow for intermittent imaging and/or distribute the measurements sparsely in space may increase the photothermal limit.

Although neuronal action potentials are the most common target of voltage imaging, our model also applies to imaging other voltage features. For instance, imaging of subthreshold voltage fluctuations might require the detection of small events, with 10× smaller signal size, β, but allow for 10× greater integration time, τ. All else being equal, Eq. (2) implies that the number of cells for which these subthreshold events could be measured would be 10× lower than the number of cells for which spikes could be measured at the same SNR. On the other hand, to image cardiac action potentials, the signal amplitude is approximately the same as for neuronal spikes, and 10× slower time resolution may be acceptable. In this case, the number of measurable cells will be ∼10× higher than for neurons.

Voltage imaging *in vivo* places stringent demands on molecular, optical, and data analysis tools. We hope that the above comprehensive analysis of these constraints will shape realistic expectations and guide efforts toward enhancing the performance of 2P voltage imaging.

## Supplementary Material



## Data Availability

The instrument control code is available at www.luminosmicroscopy.com and https://github.com/adamcohenlab/luminos-microscopy/. Data are publicly available on the DANDI archive. Scaling of GEVI fluorescence with 1P and 2P illumination intensity: https://dandiarchive.org/dandiset/000537. 1P and 2P contrast of JEDI-2P and $\text{Voltron}2_{525}$: https://dandiarchive.org/dandiset/000538. Depth dependence of fluorescence: https://dandiarchive.org/dandiset/001029.

## References

[1] Abdelfattah A. S. et al., “Bright and photostable chemigenetic indicators for extended in vivo voltage imaging,” Science 365, 699–704 (2019). SCIEAS 0036-8075 10.1126/science.aav6416 31371562

[r2] Kannan M. et al., “Dual-polarity voltage imaging of the concurrent dynamics of multiple neuron types,” Science 378, eabm8797 (2022). SCIEAS 0036-8075 10.1126/science.abm8797 36378956 PMC9703638

[3] Weber T. D. et al., “High-speed multiplane confocal microscopy for voltage imaging in densely labeled neuronal populations,” Nat. Neurosci. 26, 1642–1650 (2023). NANEFN 1097-6256 10.1038/s41593-023-01408-2 37604887 PMC11209746

[4] Villette V. et al., “Ultrafast two-photon imaging of a high-gain voltage indicator in awake behaving mice,” Cell 179, 1590–1608.e23 (2019). CELLB5 0092-8674 10.1016/j.cell.2019.11.004 31835034 PMC6941988

[5] Sims R. R. et al., “Scanless two-photon voltage imaging,” Nat. Commun. 15, 5095 (2023). NCAOBW 2041-1723 10.1038/s41467-024-49192-2 PMC11178882 38876987

[6] Liu Z. et al., “Sustained deep-tissue voltage recording using a fast indicator evolved for two-photon microscopy,” Cell 185, 3408–3425.e29 (2022). CELLB5 0092-8674 10.1016/j.cell.2022.07.013 35985322 PMC9563101

[7] Adam Y. et al., “Voltage imaging and optogenetics reveal behaviour-dependent changes in hippocampal dynamics,” Nature 569, 413–417 (2019). 10.1038/s41586-019-1166-7 31043747 PMC6613938

[r8] Fan L. Z. et al., “All-optical physiology resolves a synaptic basis for behavioral timescale plasticity,” Cell 186, 543–559.e19 (2023). CELLB5 0092-8674 10.1016/j.cell.2022.12.035 36669484 PMC10327443

[r9] Böhm U. L. et al., “Voltage imaging identifies spinal circuits that modulate locomotor adaptation in zebrafish,” Neuron 110, 1211–1222.e4 (2022). NERNET 0896-6273 10.1016/j.neuron.2022.01.001 35104451 PMC8989672

[10] Kim T. H. Schnitzer M. J., “Fluorescence imaging of large-scale neural ensemble dynamics,” Cell 185, 9–41 (2022). CELLB5 0092-8674 10.1016/j.cell.2021.12.007 34995519 PMC8849612

[11] Zong W. et al., “Large-scale two-photon calcium imaging in freely moving mice,” Cell 185, 1240–1256.e30 (2022). CELLB5 0092-8674 10.1016/j.cell.2022.02.017 35305313 PMC8970296

[12] Wilt B. A. Fitzgerald J. E. Schnitzer M. J., “Photon shot noise limits on optical detection of neuronal spikes and estimation of spike timing,” Biophys. J. 104, 51–62 (2013). BIOJAU 0006-3495 10.1016/j.bpj.2012.07.058 23332058 PMC3540268

[13] Kuhn B. Roome C. J., “Primer to voltage imaging with ANNINE dyes and two-photon microscopy,” Front. Cell. Neurosci. 13, 321 (2019). 10.3389/fncel.2019.00321 31379507 PMC6646528

[14] Charan K. et al., “Fiber-based tunable repetition rate source for deep tissue two-photon fluorescence microscopy,” Biomed. Opt. Express 9, 2304–2311 (2018). BOEICL 2156-7085 10.1364/BOE.9.002304 29760989 PMC5946790

[15] Oheim M. et al., “Two-photon microscopy in brain tissue: parameters influencing the imaging depth,” J. Neurosci. Methods 111, 29–37 (2001). JNMEDT 0165-0270 10.1016/S0165-0270(01)00438-1 11574117

[16] Zhang T. et al., “Kilohertz two-photon brain imaging in awake mice,” Nat. Methods 16, 1119–1122 (2019).1548-7091 10.1038/s41592-019-0597-2 31659327 PMC9438750

[r17] Demas J. et al., “High-speed, cortex-wide volumetric recording of neuroactivity at cellular resolution using light beads microscopy,” Nat. Methods 18, 1103–1111 (2021).1548-7091 10.1038/s41592-021-01239-8 34462592 PMC8958902

[r18] Beaulieu D. R. et al., “Simultaneous multiplane imaging with reverberation two-photon microscopy,” Nat. Methods 17, 283–286 (2020).1548-7091 10.1038/s41592-019-0728-9 32042186 PMC8590882

[r19] Meng G. et al., “Ultrafast two-photon fluorescence imaging of cerebral blood circulation in the mouse brain in vivo,” Proc. Natl. Acad. Sci. 119, e2117346119 (2022). 10.1073/pnas.2117346119 35648820 PMC9191662

[r20] Wu J. et al., “Kilohertz two-photon fluorescence microscopy imaging of neural activity in vivo,” Nat. Methods 17, 287–290 (2020).1548-7091 10.1038/s41592-020-0762-7 32123392 PMC7199528

[r21] Chamberland S. et al., “Fast two-photon imaging of subcellular voltage dynamics in neuronal tissue with genetically encoded indicators,” eLife 6, e25690 (2017). 10.7554/eLife.25690 28749338 PMC5584994

[22] Kazemipour A. et al., “Kilohertz frame-rate two-photon tomography,” Nat. Methods 16, 778 (2019).1548-7091 10.1038/s41592-019-0493-9 31363222 PMC6754705

[23] Platisa J. et al., “High-speed low-light in vivo two-photon voltage imaging of large neuronal populations,” Nat. Methods 20, 1095–1103 (2023).1548-7091 10.1038/s41592-023-01820-3 36973547 PMC10894646

[r24] Liu C. et al., “DeepVID: a self-supervised deep learning framework for two-photon voltage imaging denoising,” in Biophotonics Congr.: Biomed. Opt. 2022 (Transl., Microsc., OCT, OTS, BRAIN), Optica Publishing Group, p. BTu4C.4 (2022). 10.1364/BRAIN.2022.BTu4C.4

[r25] Eom M. et al., “Statistically unbiased prediction enables accurate denoising of voltage imaging data,” Nat. Methods 20, 1581–1592 (2023).1548-7091 10.1038/s41592-023-02005-8 37723246 PMC10555843

[r26] Lecoq J. et al., “Removing independent noise in systems neuroscience data using DeepInterpolation,” Nat. Methods 18, 1401–1408 (2021).1548-7091 10.1038/s41592-021-01285-2 34650233 PMC8833814

[27] Lecoq J. A. Podgorski K. Grewe B. F., “AI to the rescue of voltage imaging,” Cell Rep. Methods 3, 100505 (2023). 10.1016/j.crmeth.2023.100505 37426751 PMC10326374

[28] Davis H. C. et al., “Luminos,” 2023, https://github.com/adamcohenlab/luminos-microscopy/ (accessed 28 May 2024).

[29] Kimura T. et al., “Production of adeno-associated virus vectors for *in vitro* and *in vivo* applications,” Sci. Rep. 9, 13601 (2019). SRCEC3 2045-2322 10.1038/s41598-019-49624-w 31537820 PMC6753157

[30] Grienberger C. et al., “Two-photon calcium imaging of neuronal activity,” Nat. Rev. Methods Prim. 2, 67 (2022). 10.1038/s43586-021-00091-6 PMC10732251 38124998

[31] Huang Y.-L. Walker A. S. Miller E. W., “A photostable silicon rhodamine platform for optical voltage sensing,” J. Am. Chem. Soc. 137, 10767–10776 (2015). JACSAT 0002-7863 10.1021/jacs.5b06644 26237573 PMC4666802

[32] Tian H. et al., “Video-based pooled screening yields improved far-red genetically encoded voltage indicators,” Nat. Methods 20, 1082–1094 (2023).1548-7091 10.1038/s41592-022-01743-5 36624211 PMC10329731

[33] Abdelfattah A. S. et al., “Sensitivity optimization of a rhodopsin-based fluorescent voltage indicator,” Neuron 111, 1547–1563.e9 (2023). NERNET 0896-6273 10.1016/j.neuron.2023.03.009 37015225 PMC10280807

[34] Birge R. R., “Two-photon spectroscopy of protein-bound chromophores,” Acc. Chem. Res. 19, 138–146 (1986). ACHRE4 0001-4842 10.1021/ar00125a003

[35] Xu C. Webb W. W., “Measurement of two-photon excitation cross sections of molecular fluorophores with data from 690 to 1050 nm,” J. Opt. Soc. Am. B 13, 481–491 (1996). JOBPDE 0740-3224 10.1364/JOSAB.13.000481

[36] Brinks D. Klein A. J. Cohen A. E., “Two-photon lifetime imaging of voltage indicating proteins as a probe of absolute membrane voltage,” Biophys. J. 109, 914–921 (2015). BIOJAU 0006-3495 10.1016/j.bpj.2015.07.038 26331249 PMC4564826

[37] Brooks F. P. et al., “Photophysics-informed two-photon voltage imaging using FRET-opsin voltage indicators,” bioRxiv 2024.04.01.587540 (2024). 10.1126/sciadv.adp5763 PMC11708879 39772682

[38] Chien M.-P. et al., “Photoactivated voltage imaging in tissue with an archaerhodopsin-derived reporter,” Sci. Adv. 7, eabe3216 (2021). STAMCV 1468-6996 10.1126/sciadv.abe3216 33952514 PMC8099184

[39] Buijs T. J. McNaughton P. A., “The role of cold-sensitive ion channels in peripheral thermosensation,” Front. Cell. Neurosci. 14, 262 (2020). 10.3389/fncel.2020.00262 32973456 PMC7468449

[40] Kim T. et al., “Thermal effects on neurons during stimulation of the brain,” J. Neural Eng. 19, 056029 (2022).1741-2560 10.1088/1741-2552/ac9339 PMC9855718 36126646

[41] Kiyatkin E. A., “Brain temperature and its role in physiology and pathophysiology: lessons from 20 years of thermorecording,” Temperature 6, 271–333 (2019). 10.1080/23328940.2019.1691896 PMC6949027 31934603

[42] Podgorski K. Ranganathan G., “Brain heating induced by near-infrared lasers during multiphoton microscopy,” J. Neurophysiol. 116, 1012–1023 (2016). JONEA4 0022-3077 10.1152/jn.00275.2016 27281749 PMC5009202

[43] Shapey J. et al., “Optical properties of human brain and tumour tissue: an ex vivo study spanning the visible range to beyond the second near-infrared window,” J. Biophotonics 15, e202100072 (2022). 10.1002/jbio.202100072 35048541

[44] Tran A. P. Jacques S. L., “Modeling voxel-based Monte Carlo light transport with curved and oblique boundary surfaces,” J. Biomed. Opt. 25, 025001 (2020). JBOPFO 1083-3668 10.1117/1.JBO.25.2.025001 32100491 PMC7040455

[45] Wang M. et al., “Comparing the effective attenuation lengths for long wavelength in vivo imaging of the mouse brain,” Biomed. Opt. Express 9, 3534–3543 (2018). BOEICL 2156-7085 10.1364/BOE.9.003534 30338138 PMC6191617

[46] Shen Y. et al., “Engineering genetically encoded fluorescent indicators for imaging of neuronal activity: progress and prospects,” Neurosci. Res. 152, 3–14 (2020). 10.1016/j.neures.2020.01.011 31991206

[47] Vucinic D. et al., “Hybrid reflecting objectives for functional multiphoton microscopy in turbid media,” Opt. Lett. 31, 2447–2449 (2006). OPLEDP 0146-9592 10.1364/OL.31.002447 16880851 PMC2916932

[48] Engelbrecht C. J. Göbel W. Helmchen F., “Enhanced fluorescence signal in nonlinear microscopy through supplementary fiber-optic light collection,” Opt. Express 17, 6421–6435 (2009). OPEXFF 1094-4087 10.1364/OE.17.006421 19365467

[49] Cheng A. et al., “Simultaneous two-photon calcium imaging at different depths with spatiotemporal multiplexing,” Nat. Methods 8, 139–142 (2011).1548-7091 10.1038/nmeth.1552 21217749 PMC3076599

[50] Faini G. et al., “Ultrafast light targeting for high-throughput precise control of neuronal networks,” Nat. Commun. 14, 1888 (2023). NCAOBW 2041-1723 10.1038/s41467-023-37416-w 37019891 PMC10074378

[51] Bahlmann K. et al., “Multifocal multiphoton microscopy (MMM) at a frame rate beyond 600 Hz,” Opt. Express 15, 10991–10998 (2007). OPEXFF 1094-4087 10.1364/OE.15.010991 19547456

[52] Oron D. Tal E. Silberberg Y., “Scanningless depth-resolved microscopy,” Opt. Express 13, 1468–1476 (2005). OPEXFF 1094-4087 10.1364/OPEX.13.001468 19495022

[53] Lu R. et al., “Video-rate volumetric functional imaging of the brain at synaptic resolution,” Nat. Neurosci. 20, 620–628 (2017). NANEFN 1097-6256 10.1038/nn.4516 28250408 PMC5374000

[54] Wu J. Ji N. Tsia K. K., “Speed scaling in multiphoton fluorescence microscopy,” Nat. Photonics 15, 800–812 (2021). NPAHBY 1749-4885 10.1038/s41566-021-00881-0

[55] Donnert G. Eggeling C. Hell S. W., “Major signal increase in fluorescence microscopy through dark-state relaxation,” Nat. Methods 4, 81–86 (2007).1548-7091 10.1038/nmeth986 17179937

[r56] Ji N. Magee J. C. Betzig E., “High-speed, low-photodamage nonlinear imaging using passive pulse splitters,” Nat. Methods 5, 197–202 (2008).1548-7091 10.1038/nmeth.1175 18204458

[57] Jun S. et al., “Nonlinear dynamics of femtosecond laser interaction with the central nervous system in zebrafish,” Commun. Phys. 7, 161 (2024). 10.1038/s42005-024-01653-2

[58] Bloxham B. et al., “Linearly polarized excitation enhances signals from fluorescent voltage indicators,” Biophys. J. 120, 5333–5342 (2021). BIOJAU 0006-3495 10.1016/j.bpj.2021.10.028 34710379 PMC8715190

[59] Lazar J. et al., “Two-photon polarization microscopy reveals protein structure and function,” Nat. Methods 8, 684–690 (2011).1548-7091 10.1038/nmeth.1643 21725301

[60] Pulin M. et al., “Orthogonally-polarized excitation for improved two-photon and second-harmonic-generation microscopy, applied to neurotransmitter imaging with GPCR-based sensors,” Biomed. Opt. Express 13, 777–790 (2022). BOEICL 2156-7085 10.1364/BOE.448760 35284188 PMC8884218

[61] Kralj J. M. et al., “Optical recording of action potentials in mammalian neurons using a microbial rhodopsin,” Nat. Methods 9, 90–95 (2012).1548-7091 10.1038/nmeth.1782 PMC3248630 22120467

[62] Mukamel E. A. Nimmerjahn A. Schnitzer M. J., “Automated analysis of cellular signals from large-scale calcium imaging data,” Neuron 63, 747–760 (2009). NERNET 0896-6273 10.1016/j.neuron.2009.08.009 19778505 PMC3282191

[r63] Pnevmatikakis E. A. et al., “Simultaneous denoising, deconvolution, and demixing of calcium imaging data,” Neuron 89(2), 285–299 (2016). NERNET 0896-6273 10.1016/j.neuron.2015.11.037 26774160 PMC4881387

[r64] Xie M. E. et al., “High-fidelity estimates of spikes and subthreshold waveforms from 1-photon voltage imaging in vivo,” Cell Rep. 35, 108954 (2021). 10.1016/j.celrep.2021.108954 33826882 PMC8095336

[r65] Cai C. et al., “VolPy: automated and scalable analysis pipelines for voltage imaging datasets,” PLOS Comput. Biol. 17, e1008806 (2021). 10.1371/journal.pcbi.1008806 33852574 PMC8075204

[66] Buchanan E. K. et al., “Penalized matrix decomposition for denoising, compression, and improved demixing of functional imaging data,” bioRxiv 334706 (2018).

[67] Friedrich J. et al., “Multi-scale approaches for high-speed imaging and analysis of large neural populations,” PLOS Comput. Biol. 13, e1005685 (2017). 10.1371/journal.pcbi.1005685 28771570 PMC5557609

[68] Drobizhev M. et al., “Two-photon absorption properties of fluorescent proteins,” Nat. Methods 8, 393–399 (2011).1548-7091 10.1038/nmeth.1596 21527931 PMC4772972

[69] Bandmann V. et al., “Membrane capacitance recordings resolve dynamics and complexity of receptor-mediated endocytosis in Wnt signaling,” Sci. Rep. 9, 12999 (2019). SRCEC3 2045-2322 10.1038/s41598-019-49082-4 31506500 PMC6736968

[70] Denk W. Strickler J. Webb W., “Two-photon laser scanning fluorescence microscopy,” Science 248, 73–76 (1990). SCIEAS 0036-8075 10.1126/science.2321027 2321027

