# Supplementary material for: Optical constraints on two-photon voltage imaging
Source: Neurophotonics. 2024 Aug 13;11(3):035007. doi: 10.1117/1.NPh.11.3.035007 (PMC11321468; doi:10.1117/1.NPh.11.3.035007)
Supplement: Supplementary file 1 [file NPh_011_035007_SD001.pdf]

Optical constraints on two-photon voltage imaging

F. Phil Brooks III*, Hunter C. Davis*, J. David Wong-Campos, Adam E. Cohen

Department of Chemistry and Chemical Biology, Harvard University

Correspondence: cohen@chemistry.harvard.edu

Supplementary Material

Contents

1. Scaling of N_{cells}^{2P} with brightness and voltage sensitivity
2. Scaling of SNR with on and off kinetics
3. Theoretical comparison of 1P vs 2P photon efficiencies

1. Scaling of N_{cells}^{2P} with brightness and voltage sensitivity

Here we derive the dependence of the number of measurable cells on illumination intensity, fluorophore brightness, spike $\Delta F/F$, measurement bandwidth, and target SNR (Eq. 2 in the main text). We assume a point-scanning 2P illumination system which has perfect targeting to cell membranes, and which can jump between cells with zero delay. This is a best-case scenario: motion artifacts, imperfect targeting, and scanner inertia or finite slew rates further degrade the SNR. We also assume a perfect camera, which converts incident photons to counts with 100% efficiency. We convert camera counts to photons by dividing by the camera quantum efficiency (QE = ~67% @ 525 nm) and multiplying by the conversion factor (CF = 0.46 photoelectrons/digital count).

Let F (counts/s) be the rate of photons collected by a perfect detector from a single cell, let P (W) be the laser power at the focus. We define the constant for GEVI brightness, A, empirically as the proportionality between squared laser power and fluorescence signal.

$$F = A \cdot P^2, \qquad [S1]$$

where we have assumed that P is changed by adjusting total laser power, keeping laser repetition rate, pulse width, and focal and scan parameters constant.

The number of photons collected during a spike of duration τ is:

$$N_{photons} = A \cdot P^2 \cdot \tau \cdot \phi, \qquad [S2]$$

where ϕ is the fraction of the time that the laser focus intersects the cell membrane. Let β be the fractional change in fluorescence (ΔF/F) during a spike. If there is a contribution to the fluorescence from voltage-insensitive background, then β may be smaller than in the background-free case. The signal is:

$$S = \beta \cdot N_{photons} = \beta \cdot A \cdot P^2 \cdot \tau \cdot \phi \qquad [S3]$$

Assuming that the voltage change does not substantially affect the shot noise (i.e. $|\beta| \ll 1$), then the shot noise is:

$$Noise = \sqrt{N_{photons}} = \sqrt{A \cdot P^2 \cdot \tau \cdot \phi}. \qquad [S4]$$

Thus,

$$SNR = \frac{\beta \cdot N_{photons}}{\sqrt{N_{photons}}} = \beta \cdot \mathrm{P} \cdot \sqrt{A \cdot \tau \cdot \phi}. \qquad [S5]$$

This SNR calculation is applicable only when the laser is targeted to a single cell. If the laser sequentially visits N cells, then the duty cycle on each cell is $1/N$. Assuming zero transit time between cells, the number of photons collected per cell scales inversely with the number of cells:

$$N_{photons} = \frac{A \cdot P^2 \cdot \tau \cdot \phi}{N_{cells}^{2P}}. \qquad [S6]$$

Therefore,

$$SNR = \beta \cdot \mathrm{P} \cdot \sqrt{\frac{A \cdot \tau \cdot \phi}{N_{cells}^{2P}}}, \qquad [S7]$$

and

$$N_{cells}^{2P} = \frac{A \cdot \tau \cdot P^2 \cdot \beta^2 \cdot \phi}{SNR^2}. \qquad [S8]$$

Equation S8 shows the strong dependence of the number of measurable cells on the voltage sensitivity, β. The proportionality of SNR and laser power for a single cell holds true for non-scanning excitation modalities as well.[5]

2. Scaling of SNR with on and off kinetics

We use the model shown in Fig. 4a. M is the asymptotic value of the voltage step response ΔF/F at long time. We define β as the maximal value of ΔF/F for a voltage pulse of duration t, which occurs at the end of the voltage pulse. Hence:

$$\beta = \mathrm{M}\left(1 - e^{-t/\tau_{\mathrm{on}}}\right). \qquad [S9]$$

We define R_{on} as the area under the curve of ΔF/F from zero to t.

$$R_{on} = M\int_0^t (1 - e^{-t'/\tau_{on}})\, dt' = M\left(t - \tau_{on}\left(1 - e^{-t/\tau_{on}}\right)\right). \quad [S10]$$

Similarly, the fluorescence decay after the voltage pulse is:

$$\frac{\Delta F_{off}}{F}(t') = \beta e^{-t'/\tau_{off}}. \quad [S11]$$

The area under this portion of the response curve is:

$$R_{off} = \beta\int_0^\infty e^{-t'/\tau_{off}}\, dt' = \beta\tau_{off} = M\tau_{off}\left(1 - e^{-t/\tau_{on}}\right). \quad [S12]$$

The total response, R, is the sum of these two parts:

$$R = M\left(t + (\tau_{off} - \tau_{on})\left(1 - e^{-t/\tau_{on}}\right)\right). \quad [S13]$$

To express SNR, we recognize that R takes the place of $\beta \cdot \tau$ in Eq. S3.

$$S = \beta \cdot \tau \cdot A \cdot P^2 \cdot \phi = R \cdot F \cdot \phi. \quad [S14]$$

We also set the total integration time, τ, in Eq. S4 to be $t + \tau_{off}$

$$Noise = \sqrt{A \cdot P^2 \cdot \tau \cdot \phi} = \sqrt{F \cdot \phi(t + \tau_{off})}\ . \quad [S15]$$

We combine to get:

$$SNR = \frac{RF\phi}{\sqrt{F\phi(t+\tau_{off})}}. \quad [S16]$$

3. Theoretical comparison of 1P vs 2P photon efficiencies

We use the properties of JEDI-2P as an exemplary GEVI which works under both 1P and 2P excitation. We assume that the 1P and 2P absorption cross sections of the JEDI-2P chromophore are the same as for eGFP. While this assumption may not be exact, modest variations in these cross sections will not change the conclusion that 2P voltage imaging requires ~10^4-fold more power per cell, compared to 1-P voltage imaging.

First, we estimate the per-molecule excitation rate under 1P excitation. To achieve a per-cell detected digital count rate of 1.5×10^7 s^{-1} (equivalent to 10^7 impinging photons/s), required a mean per-cell laser power of 9.6×10^{-7} W, or equivalently an illumination intensity of ~1 W/cm^2 (Fig. 1; assuming a HEK cell is approximately 10 μm diameter). This intensity is in the middle of the range used for *in vivo* 1P voltage imaging: recordings of Voltron2 in flies used 200 – 1100 mW/cm^2,[33] while high-speed recordings of PV cells in mice used up to 14 W/cm^2.[33]

The decadal molar absorption coefficient of eGFP is ε = 45,000 M^{-1} cm^{-1}.[68] The per-molecule excitation rate is:

$$\Gamma_{1P} = I\epsilon \frac{\lambda}{h\,c} \frac{10^3 \ln 10}{N_A}, \qquad [S17]$$

where I (W/cm^2) is the incident intensity, ϵ (M^{-1} cm^{-1}) is the decadal molar absorption coefficient, λ (m) is the wavelength, h (6.63×10^{-34} J s) is Planck's constant, c (3×10^8 m/s) is the speed of light, and N_A (6.02×10^{23} mol^{-1}) is Avogadro's number. We assume λ = 488 nm and find that at I = 1 W/cm^2, $\Gamma_{1P} = 430\ s^{-1}$; at 10 W/cm^2, $\Gamma_{1P} = 4300\ s^{-1}$.

Assuming an overall 10% total light collection efficiency (reasonable for a high NA optical system), 10^7 collected photons/s corresponds to 10^8 emitted photons/s. At a per-molecule emission rate of 430 s^{-1}, this implies 2.3×10^5 molecules/cell. At a typical HEK cell membrane surface area of 1000 μm^2,[69] the density of reporters is 230 μm^{-2}.

We now estimate the 2P power needed to match the emitted count rate of 10^8 s^{-1}. The probability that a fluorophore is electronically excited by a single pulse from a 2P optical system is:[70]

$$P_{2P} = \frac{\sigma_2 P_{avg}^2}{\tau_{2P} f_{2P}^2} \left(\frac{[NA]^2}{2\hbar c\lambda}\right)^2, \qquad [S18]$$

where σ_2 is the 2P absorption cross section (m^4 s), P_{avg} (W) is the time-average power from the laser, τ_{2P} (s) is the pulse duration, f_{2P} is the laser repetition frequency, NA is the objective lens numerical aperture, and $\hbar$, c, and λ are as above. The per-molecule rate of excitation is $\Gamma_{2P} = f_{2P} P_{2P}$.

We assume parameters typical of a 2P imaging experiment: NA = 1, λ = 920 nm, τ_{2P} = 200 fs, f_{2P} = 80 MHz. The 2P absorption cross section of eGFP is σ_2 = 39 GM (39×10^{-58} m^4 s).[68]

The brightest signal from the cell arises when the laser focus intersects an equatorial membrane, so the optical axis lies in the plane of the membrane, as in Fig. 4c. In this case the membrane area that is optically excited is approximately $A_{2P} = w_0 b$, where w_0 is the waist of the Gaussian focus and b is the depth of focus. The focus waist is approximately $w_0 = \frac{\lambda}{2NA}$, and the depth of focus is $b = 2\pi w_0^2 n/\lambda$, where n = 1.33 is the index of refraction. This estimate yields A_{2P} ~ 1 μm^2, implying that ~230 reporter molecules are in the 2P focus. To achieve a total emitted photon rate of 10^8 s^{-1} then implies a per-molecule emission rate of $\Gamma_{2P} = 4.3\times10^5$ s^{-1}. The time-average laser power to achieve this count rate is 8 mW, 10^4-fold higher than the 1P power to achieve the same count rate. In the minimal SNR limit of 2.5×10^5 detected photons/s/cell (corresponding to 2.5×10^6 emitted photons/s/cell), the minimum time-average 2P power per cell is 0.4 mW (assuming ϕ = 1).
